# Cognitive aging in rural communities: preliminary memory characterization of a community cohort from Southern Nevada

**DOI:** 10.3389/frdem.2023.1236039

**Published:** 2023-08-24

**Authors:** Justin B. Miller, Christina G. Wong, Jessica Z. K. Caldwell, Jessica Rodrigues, Shehroo Pudumjee, Samantha E. John, Aaron Ritter

**Affiliations:** ^1^Cleveland Clinic Lou Ruvo Center for Brain Health, Las Vegas, NV, United States; ^2^Department of Brain Health, University of Nevada, Las Vegas, NV, United States; ^3^Memory & Cognitive Disorders Program, Hoag Hospital, Pickup Family Neurosciences Institute, Newport Beach, CA, United States

**Keywords:** rural health, Alzheimer's disease, geographic disparities, aging, memory, dementia, neighborhood disadvantage

## Abstract

**Introduction:**

Rural-dwelling older adults face unique health challenges that may increase risk for Alzheimer's disease and dementia but are underrepresented in aging research. Here, we present an initial characterization of a rural community cohort compared to an urban cohort from the same region.

**Methods:**

Adults over age 50 living in a non-metropolitan area are clinically characterized using the Uniform Data Set, enriched with additional measures of verbal and non-verbal memory measures. Neighborhood disadvantage is also assessed. Clinical and cognitive differences between cohorts were explored after stratifying by cognitive impairment.

**Results:**

Between group comparisons found that rural-dwellers demonstrated better verbal memory than urban-dwellers on primary indices of learning, recall, and recognition, with small to medium effects in overall comparisons. When stratified by impairment, rural-urban differences were notably larger among cognitively normal individuals. Within-group comparisons found that the magnitude of impairment between cognitively normal and impaired groups was greater among rural-dwellers compared to urban-dwellers. No differences in non-verbal memory or overall clinical status were found, and there were no effects of neighborhood disadvantage on any cognitive measure.

**Discussion:**

Living in a rural community presents a complex set of contextual factors that for some, may increase risk for dementia. In this study, we found small to moderate memory advantages for rural-dwellers, leaving open the possibility that late-life rural living may be advantageous for some and promote resilience. Additional prospective research is critically needed to better understand the factors that influence aging outcomes in this underrepresented population.

## 1. Background

Older adults living in rural areas are thought to face significantly greater risk for Alzheimer's disease and related dementias (AD/RD) (Weden et al., 2018; Rahman et al., 2021; Liu et al., 2022) and worse clinical outcomes than those living in urban communities (Abner et al., 2016; Weden et al., 2018; Rahman et al., 2020; Cato et al., 2022). Despite these geographic health disparities, rural individuals are underrepresented in aging research, and a comprehensive understanding of the factors contributing to urban-rural disparities in AD/RD remains unclear. As over 40% of the global population live in rural areas (United Nations, Department of Economic and Social Affairs, Population Division, 2019), and rural communities tend to have a higher proportion of older residents compared to urban communities (Kinsella, 2001) the need for more research and services dedicated to healthy rural aging is clear.

A complex interplay between individual demographic factors, health behaviors, and the sociocultural environment likely affects rural aging outcomes. AD/RD research often focuses on individual risk and protective factors, whereas contextual factors have historically received less attention. Neighborhood disadvantage (ND), for example, is a multidimensional social determinant of health (SDoH) that reflects regional education, poverty, employment, housing, crime and safety, and food insecurity that could account for the urban-rural differences in AD/RD outcomes. Rural areas are typically associated with greater socioeconomic disadvantage as compared to urban areas, which contributes to disparities in overall mortality rates (Long et al., 2018). As measured by the Area Deprivation Index (ADI) (Singh, 2003; Kind et al., 2014; Kind and Buckingham, 2018), ND has been associated with AD-specific patterns of neurodegeneration (Hunt et al., 2020, 2021) and AD/RD neuropathological changes, including accumulation of amyloid plaques (Powell et al., 2020) and neurofibrillary tangles (Powell et al., 2022). Greater ND is also associated with an increased risk of cognitive impairment and overall dementia risk (Pase et al., 2022; Vassilaki et al., 2022). Most of this literature, however, has investigated small pockets of disadvantage within urban communities, and it is unknown if these findings generalize to rural communities.

Barriers to care including transportation barriers and healthcare professional shortages in geographically isolated areas may also contribute to AD/RD disparities among rural residents. Notably, health care access and quality are important social determinants of health not captured by ND scores. Lack of access to specialty care may be the most influential factor related to reception of diagnosis and treatment. Retrospective data from Medicare claims data reflecting U.S. trends show that older rural-dwelling adults are more likely to be misdiagnosed, if they are diagnosed at all (Abner et al., 2016; Rahman et al., 2021). Other research indicates that patients with AD/RD are more likely to be diagnosed by their primary care provider rather than a specialist (Xu et al., 2022). Furthermore, those who are diagnosed are often in advanced stages (Abner et al., 2016; Rahman et al., 2021), which may lead to shorter survival periods. Issues with regional distribution of medications, lower household income, and less access to healthcare are also factors that affect treatment of AD/RD in rural areas (Zhang et al., 2017; Li et al., 2022).

Despite the many challenges related to AD/RD diagnosis and care in rural communities, there have been very few prospective studies with the primary intention of characterizing cognitive aging and brain health among those living in rural areas. The Nevada Exploratory Alzheimer's Disease Research Center (NVeADRC) seeks to address these gaps by prospectively enrolling a community-based sample of older adults living in rural areas in the Southwestern United States and following them over time. Cognitive functioning remains a central component in the clinical evaluation, diagnosis, treatment planning, and disease monitoring of AD/RD, and in this paper, we present our initial characterization of memory in our cohort in comparison to a harmonized urban cohort followed in the same geographic region. As it has been suggested that rural living increases risk for AD/RD, we hypothesized that differences in cognition between urban and rural dwelling adults will emerge, with rural dwelling older adults performing worse on memory testing, especially earlier in the disease course or before cognitive impairment has become fully manifest (e.g., mild cognitive impairment). We also hypothesized that larger effects will be evident among those with more advanced disease (e.g., dementia), consistent with an accelerated prospective decline. Second, we explored the influence of ADI on cognition. We expected greater levels of disadvantage in our rural cohort, which we predicted would account for a significant proportion of variance in cognitive outcomes when assessed across the urban and rural continuum.

## 2. Materials and methods

### 2.1. Participants

#### 2.1.1. Rural cohort

The NVeADRC is actively enrolling community-dwelling adults over the age of 50, who maintain a primary and current residence in a non-metropolitan area in the broader Desert Southwest Region (DSR) surrounding Las Vegas, Nevada. For purposes of this study, determinations of geographic eligibility were made using the Rural Urban Commuting Area (RUCA) codes published by the United States Department of Agriculture (USDA) (USDA ERS, 2020), which are frequently used in rural health research (Hart et al., 2005; Danek et al., 2022; Shora et al., 2023). Each individual's primary residence was assigned a RUCA code based on the full 5-digit zip code, with RUCA codes ≥ 4 considered rural. Individuals must be proficient in English and willing to participate in a longitudinal research program that entails sharing of de-identified cognitive, behavioral, medical, and genetic data, as well as biospecimens and related biomarker data with the scientific community. They must also have a reliable study partner who is able to provide collateral information at each visit. Those with an established neurological disorder other than AD or related dementia (e.g., large vessel stroke, traumatic brain injury, and epilepsy), unstable medical conditions, history of major psychiatric disorder (e.g., schizophrenia), or active substance abuse or dependence are ineligible. The primary method of recruitment has been direct community outreach and engagement through education events, memory screenings, an online registry, and word-of-mouth. Individuals meeting eligibility requirements who are followed clinically at the Cleveland Clinic Lou Ruvo Center for Brain Health (LRCBH) were also invited to enroll, though our primary methods of recruitment have been community based.

#### 2.1.2. Urban cohort

To facilitate comparisons across the urban-rural continuum, data collected from urban-dwelling older adults followed by the Center for Neurodegeneration and Translational Neuroscience (CNTN) were also used; details of this cohort have been previously published (Ritter et al., 2018). In brief, the overarching design of the CNTN parallels the NVeADRC in that it is a longitudinal, observational study of brain aging, Alzheimer's disease, and related dementias. The CNTN and NVeADRC protocols have been aligned to the extent possible, with many common or harmonized, data elements. Aside from recruiting individuals who live in urban or suburban areas, defined by RUCA codes of < 4, individuals in the CNTN have primarily been recruited from the clinical population at the LRCBH.

### 2.2. Measures

#### 2.2.1. Demographic data

For each participant, primary demographic variables of interest include age, sex, education, race, and ethnicity. We also identified the RUCA code and ADI scores associated with each participant's current primary residence. For ADI, we used state decile rankings based on the individual's primary state of residence. For the RUCA codes, higher values are considered more rural and higher ADI values are associated with greater neighborhood disadvantage. Generally, more rural areas are less populated and under-resourced, with lower SES and lower education, thereby leading to greater neighborhood disadvantage as measured by the ADI.

#### 2.2.2. Neuropsychological testing

The primary battery of neuropsychological tests used in the NVeADRC and CNTN includes the full Uniform Data Set, 3.0 (UDS3; see Weintraub et al., 2018 for a full listing of UDS3 cognitive measures), with additional measures of verbal list learning (Rey Auditory Verbal Learning Test; RAVLT; Schmidt, 1996), non-verbal learning and memory (Brief Visuospatial Memory Test, Revised; BVMT-R; Benedict, 1997), and an estimate of premorbid intelligence (Wide Range Achievement Test, 4th Edition Reading Subtest; WRAT-4; Wilkinson and Robertson, 2006). As the focus of the present paper is specifically on memory, analyses focused on the primary measures generated by standard administration of the RAVLT and BVMT-R including total acquisition (e.g., sum of learning trials), delayed recall, and total recognition. The Geriatric Depression Scale (GDS; Sheikh and Yesavage, 1986) is also administered as part of standard assessment, which was used to characterize the burden of depressive symptoms experienced by patients.

### 2.3. Procedure

The NVeADRC and the CNTN are longitudinal, observational cohort studies that do not include any active intervention. The NVeADRC began enrolling participants in February, 2021 and the CNTN began in enrolling in April, 2016; both continue to actively enroll. Individuals meeting enrollment criteria are seen for a comprehensive baseline visit and annually thereafter at LRCBH in Las Vegas, NV. This visit includes a medical examination, blood draw for both clinical labs and blood-based biomarker characterization, neuropsychological testing, informant interview, and structural brain MRI. All study procedures are repeated annually, except for imaging, which is repeated biannually.

After each visit, participants are assigned a research diagnosis of cognitively normal (CN), mild cognitive impairment (MCI), or dementia through consensus review by a panel of licensed clinicians including neurologists, neuropsychologists, and advanced practice providers. For some individuals in the CNTN, diagnosis was established by an individual clinician, though most were reviewed via consensus conference. Information considered in diagnostic decision making included neuropsychological test data, clinical exam, medical history, structural MRI with volumetrics, and informant reports of clinical course and functional independence. For those meeting criteria for a neurocognitive disorder, a suspected etiology was then identified. Where available, amyloid PET imaging was considered to identify suspected etiology after a cognitive diagnosis was rendered.

Both the NVeADRC and CNTN protocols have been reviewed and approved by the Institutional Review Board at Cleveland Clinic, and all study procedures are performed in accordance with the ethical standards set forth by the Declaration of Helsinki and its later amendments.

### 2.4. Analyses

The overarching comparisons of interest were to better understand group differences in memory functioning between urban and rural dwelling older adults. As such, primary group membership was defined based on study cohort, reflecting geographic region (e.g., rural/ADRC vs. urban/CNTN). We further stratified analyses by cognitive status. Given the small sample of individuals with dementia, we collapsed those with MCI and dementia into a single cognitively impaired group, creating 4 primary comparison groups: (1) impaired rural; (2) impaired urban; (3) unimpaired rural; (4) unimpaired urban. Differences in demographic variables were explored using analysis of variance (ANOVA) for continuous variables (e.g., age, education, and premorbid intelligence) or chi-square for categorical variables (e.g., sex, race, and ethnicity). Any group differences in demographics would be used as covariates in subsequent analyses. All analyses used raw memory test scores.

For our primary analyses assessing the main effects of rural residence on cognition, we conduced ANOVA or ANCOVA, as appropriate, using the primary outcome measure from the RAVLT or BVMT-R as the dependent variable with urban/rural group membership as the primary independent variable, along with any differences in demographics as covariates. Within each geographical cohort, comparisons between cognitive groups would be circular as group membership is defined on the basis of cognitive test performance; however, effect sizes between impaired and unimpaired groups were calculated and compared in order to approximate the relative degree to which cognitive impairment manifests in rural communities relative to urban communities. Effect sizes between urban and rural groups were calculated for the overall sample, as well as between impaired and unimpaired groups for the rural and urban cohorts separately. As these analyses represent a preliminary comparison and initial characterization, we did not adjust thresholds for statistical significance, presenting effect sizes for each comparison (where applicable).

Secondary analyses explored the relationship between ADI and cognition in the combined overall cohort, and the urban and rural cohorts separately. Using each primary memory outcome as the dependent variable, we fit several linear regression models using demographic information and ADI state decile rank as predictors to understand the relative influence of neighborhood disadvantage on cognition, over and above demographic information. Models were fit without cognitive status included to avoid circularity, and primary memory indices reflecting learning, delayed recall, and recognition were included from both verbal and non-verbal memory tasks as predictors, along with age, sex, education, and ADI.

## 3. Results

### 3.1. Sample characteristics

Demographic characteristics for the overall sample and within the impaired and unimpaired groups are presented in Table 1. The overall rural sample (*n* = 81) was primarily non-Hispanic (96.3%) white (90%), women (63.0%), with a mean age of 70.7 ± 6.7 years and 15.3 ± 2.5 years of education. Approximately half of the rural sample was cognitively normal (51.9%), with a mean age of 69.2 ± 5.9 years and 15.7 ± 2.5 years of education (range = 12–20 years) while the impaired rural group (43.2%; MCI *n* = 25, Dementia *n* = 10) had a mean age of 72.5 ± 7.5 years and 14.8 ± 2.4 years of education; 4 individuals were missing diagnosis information at the time of analysis as they had not yet been reviewed by consensus panel and were excluded from analyses. Women made up 71.4% of the cognitively normal rural sample and 48.6% of the impaired rural sample.

**Table 1:** Descriptive demographic and clinical data by group.

	**Overall**		**Cognitively normal**		**Impaired**	
	**Rural**	**Urban**	**Rural**	**Urban**	**Rural**	**Urban**
	***n*** = **81**	***n*** = **129**	***n*** = **42**	***n*** = **57**	***n*** = **35**	***n*** = **47**
Age (years)	70.7 (6.7)	71.5 (7.2)	69.2 (5.9)	70.0 (7.0)	72.5 (7.5)	74.0 (6.5)
Education (years)	15.3 (2.5)	15.8 (2.5)	15.7 (2.5)	16.1 (2.6)	14.8 (2.4)	16.0 (2.3)
ADI (state decile)	5.9 (2.6)	3.5 (2.3)	5.4 (2.7)	3.5 (2.4)	6.5 (2.4)	3.4 (2.1)
Sex (%female)	63.0%	46.5%	71.4%	54.4%	48.6%	34.0%
Ethnicity (%Hispanic)	3.7%	8.5%	2.3%	8.8%	5.7%	10.6%
**Race**						
White	90.0%	90.0%	83.3%	87.7%	97.0%	95.7%
Black	3.7%	3.1%	7.1%	3.5%	0.0%	0.0%
Asian	2.5%	5.4%	4.8%	8.8%	0.0%	4.3%
Other	3.8%	1.5%	4.8%	0.0%	3.0%	0.0%
MoCA	24.2 (4.3)	24.4 (3.4)	26.6 (2.3)	26.5 (2.6)	21.7 (4.4)	21.9 (3.4)
CDR sum of boxes	1.1 (1.9)	1.4 (1.9)	0.3 (0.7)	0.4 (0.8)	2.1 (2.5)	2.5 (2.1)

4 missing diagnosis in Rural; 25 missing diagnosis in Urban.

ADI, Area Deprivation Index; MoCA, Montreal Cognitive Assessment; CDR, Clinical Dementia Rating Scale.

The overall urban sample (*n* = 129) was 46.5% women and primarily non-Hispanic (91.5%) white (90.0%), with a mean age of 71.5 ± 7.2 years and 15.8 ± 2.5 years of education. As with our rural cohort, most of the urban cohort was classified as cognitively normal (44.1%) with a mean age of 70.0 ± 7.0 years and 16.1 ± 2.6 years of education (range = 9–20 years). The impaired urban sample (36.4%; MCI *n* = 38, dementia *n* = 9) had a mean age of 74.0 ± 6.5 years and 16.0 ± 2.3 years of education; 25 individuals were missing diagnosis information at the time of analysis. Women made up 54.4% of the cognitively normal urban sample and 34.0% of the impaired urban sample.

There were no differences in age, education, race, ethnicity, or the proportion of impaired individuals between the overall geographic groups, though there was a higher proportion of women in the rural cohort (*X*^2^ = 4.62, *p* < 0.05) relative to the urban cohort. In the cognitively normal group, there were no differences in any demographic variable between urban and rural groups. Within the cognitively impaired group, there was no significant difference in demographic variables either, except for education, which was significantly lower among rural dwelling adults compared to urban dwelling adults [*F*_(1,80)_ = 5.36, *p* < 0.05, Cohen's *d* = 0.51]. Regarding neighborhood characteristics, the mean RUCA codes were 1.2 ± 1.0 and 4.0 ± 1.2 for the urban and rural cohorts, respectively, which aligns with our enrollment criteria. Overall, the rural cohort was significantly more disadvantaged than our urban cohort, as measured by the ADI [rural ADI = 5.9 ± 2.6; urban ADI = 3.5 ± 2.3; *F*_(1,182)_ = 41.93, *p* < 0.001, *d* = 0.98], with 32.1% of rural participants living in severely disadvantaged neighborhoods (ADI ≥ 8) and 12.3% living in the most disadvantaged area (ADI = 10). In the urban cohort, 7.8% of people were living in a severely disadvantaged neighborhood and only 1 individual was living in the most disadvantaged ADI level. Within the urban cohort, there were no differences in neighborhood disadvantage between impaired (ADI = 3.4 + 2.1) and unimpaired groups (ADI = 3.5 + 2.4). Within the rural cohort, the difference in neighborhood disadvantage between impaired (ADI = 6.5 + 2.4) and unimpaired groups (ADI = 5.4 + 2.7) approached significance [*F*_(1,75)_ = 3.89, *p* = 0.05, *d* = 0.43].

### 3.2. Clinical characteristics

A summary of general cognitive and functional screening measures is presented in Table 1. Cognitive screening, as assessed by the Montreal Cognitive Assessment (MoCA; Nasreddine et al., 2005) and functional status, as measured by the Clinical Dementia Rating Sum of Boxes (CDR; Morris, 1993), followed an expected pattern, with lower scores evident in the impaired groups relative to the unimpaired group. There were no differences in MoCA Total Score or CDR Sum of Boxes between the overall urban and rural groups or when stratified by impairment status. Within the rural cohort, the effect of cognitive impairment as measured by the MoCA between impaired and unimpaired groups was 1.40 (Cohen's *d*), whereas in the urban group was 1.52. Measures of subjective cognitive decline were only available for the rural cohort. Approximately one-third of participant's subjective experiences differed from objective testing, with 33% of those who were found to have normal cognition reporting subjective experiences of decline and 32% of those diagnosed with cognitive impairment denying experiences of decline. There were no differences in the proportion of individuals with a subjective memory complaint in the cognitive status group comparisons within the rural cohort (*X*^2^ = 2.89, *p* = 0.08).

In regard to mood, 25% of the urban endorsed a history of depression within the past 2 years and 28.6% reported a history of anxiety. Approximately 28.2% of the rural sample reported a history of depression and 26.7% reported a history of anxiety. There were no differences between the overall urban and rural cohorts in the proportion of individuals self-reporting depression and anxiety, and there were no differences in current depressive symptoms as measured by the GDS.

### 3.3. Verbal learning and memory

Summary statistics for both verbal and non-verbal learning and memory tests are presented in Tables 2, 3, respectively. For total acquisition of information across all individual learning trials (RAVLT Trials 1−5 Total), after accounting for the effects of age, sex, and education, there were significant main effects for both geographic cohort [*F*_(1,157)_ = 11.90, *p* < 0.001] and impairment group [*F*_(1,157)_ = 40.75, *p* < 0.001], such that the urban group performed significantly worse than the rural group and the impaired group performed significantly worse than the unimpaired group; however, there was no interaction between impairment group and geographic cohort after accounting for demographics. As expected, cognitive status was associated with large effect sizes in total acquisition differences for both the urban (*d* = 1.23) and rural cohorts (*d* = 1.65). Short-delay free recall (RAVLT Trial 6) significantly differed between groups, with main effects for impairment status [*F*_(1,157)_ = 62.71, *p* < 0.001] and geographic cohort [*F*_(1,157)_ = 5.65, *p* < 0.05]. A significant interaction was also found [*F*_(1,157)_ = 6.68, *p* < 0.05] such that the rural group performed better than the urban group in the overall comparisons (*d* = 0.13) and the cognitively normal group (*d* = 0.57); however, within the impaired group, the rural cohort performed significantly worse (*d* = 0.28). As with the learning trials, the effects of cognitive impairment were more pronounced in the rural group (*d* = 2.00) than the urban group (*d* = 1.20). After a longer delay, a similar pattern emerged with significant main effects of impairment group [*F*_(1,157)_ = 57.16, *p* < 0.001] and geography [*F*_(1,157)_ = 14.20, *p* < 0.001], but there was no interaction between impairment status and geography. For delayed recall, the effects of cognitive impairment on memory testing were notably larger for the rural cohort (*d* = 2.06) than the urban cohort (*d* = 1.45). Comparisons between groups on overall recognition accuracy (RAVLT Recognition % Correct) showed significant main effects of both geography [*F*_(1,157)_ = 5.54, *p* < 0.05] and impairment status [*F*_(1,157)_ = 36.92, *p* < 0.001], but no interaction was found. The effects of impairment in the urban cohort (*d* = 0.80) were half the size of effect in the rural cohort (*d* = 1.62).

**Table 2:** Rey auditory verbal learning test performance by group.

	**Overall**					**Cognitively normal**					**Impaired**				
	**Rural (*****n*** = **78)**		**Urban (*****n*** = **101)**		***d***	**Rural (*****n*** = **42)**		**Urban (*****n*** = **49)**		***d***	**Rural (*****n*** = **33)**		**Urban (*****n*** = **40)**		***d***
	***M***	***SD***	***M***	***SD***		***M***	***SD***	***M***	***SD***		***M***	***SD***	***M***	***SD***	
Trial 1	4.81	1.90	4.48	1.46	0.20	5.43	1.86	4.82	1.32	0.38	3.94	1.52	3.88	1.49	0.04
Trial 2	7.81	2.64	6.54	2.18	0.52	9.10	2.30	7.47	1.93	0.77	6.24	1.89	5.38	2.08	0.43
Trial 3	9.24	2.87	8.02	2.48	0.45	10.71	2.39	8.98	2.19	0.75	7.33	2.16	6.65	2.35	0.30
Trial 4	9.91	3.34	8.79	2.66	0.37	11.60	2.87	10.02	2.29	0.61	7.70	2.40	7.20	2.50	0.20
Trial 5	10.68	3.26	9.47	2.97	0.39	12.43	2.41	10.69	2.22	0.75	8.39	2.60	7.68	3.06	0.25
Trials 1−5 total	42.45	12.54	37.30	10.32	0.45	49.26	9.97	41.98	7.94	0.81	33.61	9.03	30.77	10.21	0.29
Learning over trials	18.41	8.83	14.92	8.09	0.41	22.12	7.80	17.90	7.73	0.54	13.91	7.46	11.40	8.17	0.32
List B	4.74	2.37	4.28	1.70	0.22	5.62	2.06	4.88	1.47	0.41	3.48	2.22	3.33	1.58	0.08
Trial 6	7.67	4.62	7.11	3.67	0.13	10.50	3.05	8.80	2.91	0.57	3.94	3.49	4.92	3.53	−0.28
Delayed recall	7.73	4.70	5.58	4.26	0.48	10.67	2.99	7.82	3.60	0.86	3.97	3.49	2.62	3.56	0.38
Recognition hits	12.22	3.59	11.62	3.26	0.17	14.12	1.23	12.63	2.27	0.82	9.66	4.14	9.80	3.86	−0.03
Recognition false pos.	1.08	1.17	1.57	1.89	−0.31	0.83	1.06	1.27	2.05	−0.27	1.41	1.21	1.82	1.66	−0.28
Recognition % correct	87%	13%	83%	14%	—	94%	5%	88%	11%	—	77%	14%	77%	16%	—
Short term retention	65%	31%	71%	27%	—	83%	14%	82%	19%	—	42%	31%	58%	31%	—
Long term retentions	65%	32%	52%	35%	—	85%	15%	70%	25%	—	41%	31%	27%	32%	—
Memory efficiency	1.56	0.64	1.34	0.65	0.34	1.96	0.28	1.66	0.44	0.81	1.06	0.60	0.88	0.67	0.28

d, Cohen's d.

**Table 3:** Brief visuospatial memory test, revised performance by group.

	**Overall**					**Cognitively normal**					**Impaired**				
	**Rural (*****n*** = **76)**		**Urban (*****n*** = **101)**		***d***	**Rural (*****n*** = **41)**		**Urban (*****n*** = **49)**		***d***	**Rural (*****n*** = **33)**		**Urban (*****n*** = **40)**		***d***
	***M***	***SD***	***M***	***SD***		***M***	***SD***	***M***	***SD***		***M***	***SD***	***M***	***SD***	
Trial 1	4.21	2.17	3.38	2.28	0.37	5.17	1.87	4.08	2.47	0.50	2.94	1.81	2.50	1.68	0.25
Trial 2	6.71	2.97	5.88	2.99	0.28	8.37	2.28	7.41	2.59	0.39	4.47	2.20	4.05	2.47	0.25
Trial 3	8.03	3.12	7.32	3.37	0.22	9.80	2.05	9.10	2.50	0.31	5.81	2.63	5.12	3.06	0.24
Trials 1−3 total	18.95	7.73	16.57	8.04	0.30	23.34	5.45	20.59	6.89	0.44	13.22	6.10	11.68	6.70	0.24
Learning over trials	3.82	2.07	4.08	2.30	−0.12	4.63	1.96	5.08	2.14	−0.22	2.88	1.77	2.85	1.93	0.02
Delayed recall	7.27	3.61	6.40	3.83	0.23	9.44	2.13	8.45	2.87	0.39	4.64	3.14	3.88	3.51	0.23
% Retention	85.6%	35.2%	76.7%	33.7%	—	96.4%	15.8%	90.9%	18.5%	—	74.3%	46.0%	58.8%	40.7%	—
Recognition hits	5.43	0.91	5.54	0.82	−0.13	5.76	0.54	5.84	0.43	−0.16	5.00	1.12	5.12	1.07	−0.11
Recognition false positive	0.21	0.57	0.45	0.91	−0.32	0.00	0.00	0.04	0.20	−0.28	0.45	0.79	1.00	1.20	−0.54
Recognition discriminability	5.22	1.24	5.10	1.40	0.09	5.76	0.54	5.80	0.50	−0.08	4.55	1.56	4.12	1.67	0.27

d, Cohen's d; all test performance values reflect unadjusted raw scores.

### 3.4. Non-verbal learning and memory

Total acquisition across learning trials for non-verbal information (BVMT Total Trials 1–3) significantly differed between impaired and unimpaired groups [*F*_(1,155)_ = 35.48, *p* < 0.01] but did not differ between urban and rural groups [*F*_(1,155)_ = 3.80, *p* = 0.05] after accounting for the effects of age, sex, and education. There was no interaction between impairment status and geographical cohort. Comparing the impaired and unimpaired groups in each cohort finds larger effects for the rural group (*d* = 1.75) than the urban group (*d* = 1.31). Learning over trials, delayed recall, and delayed recognition followed the same pattern, with no differences observed between urban and rural groups, and no interaction between cohort and impairment status, though main effects of impairment status were found, as expected. Delayed recall effect sizes were again larger in the rural cohort (*d* = 1.79) than the urban cohort (*d* = 1.43).

### 3.5. Neighborhood disadvantage

To assess the relative influence of neighborhood disadvantage on cognitive function, several linear regression models were fit using age, education, sex and ADI to predict each of the primary memory variables; model summary statistics are presented in Table 4. For the combined cohort, each regression model fit was significant, except for RAVLT recognition; however, the total proportion of variance accounted for by each model was small (*R*^2^ = 0.07–0.19). Age was a significant predictor for all variables, as expected, and sex was associated with RAVLT learning and delayed recall. Education was only associated with RAVLT delayed recall. ADI was not a significant predictor in any of the models fit after demographic variables were included. Within the urban cohort, models predicting RAVLT learning and delayed recall, and BVMT learning were the only significant models. Age was the only significant predictor of BVMT learning and sex was the only significant predictor of RAVLT delayed recall. None of the individual predictors of RAVLT learning were significant.

**Table 4:** Linear regression models including demographics and ADI.

		**Standardized coefficients**			
	***R* ^2^**	**Age**	**Sex**	**Education**	**ADI**
**Overall**					
RAVLT T1−5	0.18	−0.22	0.29	0.15	0.03
RAVLT delayed recall	0.19	−0.24	0.25	0.20	0.09
RAVLT recognition	0.05	−0.09	0.17	0.02	−0.04
BVMT learning	0.15	−0.32	0.09	0.14	−0.01
BVMT delayed recall	0.12	−0.32	0.06	0.12	0.01
BVMT recognition	0.07	−0.25	0.05	0.07	−0.04
**Rural**					
RAVLT T1−5	0.25	−0.18	0.27	0.17	−0.19
RAVLT delayed recall	0.27	−0.31	0.18	0.21	−0.16
RAVLT recognition	0.14	−0.14	0.16	0.11	−0.20
BVMT learning	0.20	−0.31	0.07	0.18	−0.18
BVMT delayed recall	0.17	−0.38	−0.01	0.15	−0.06
BVMT recognition	0.14	−0.36	−0.08	0.06	−0.10
**Urban**					
RAVLT T1−5	0.14	−0.22	0.22	0.12	0.11
RAVLT delayed recall	0.16	−0.13	0.26	0.21	0.20
RAVLT recognition	0.02	−0.02	0.11	−0.05	0.02
BVMT learning	0.13	−0.31	0.07	0.09	0.07
BVMT delayed recall	0.10	−0.27	0.09	0.10	0.01
BVMT recognition	0.06	−0.15	0.15	0.08	−0.01

ADI, Area Deprivation Index; RAVLT Recognition, Percent Correct.

All models fit predicting memory outcomes in the rural cohort were significant, with larger proportions of variance accounted for than models fit in the urban cohort. Age was a significant predictor in all models, except for RAVLT learning and recognition. As was the case in the overall cohort, sex was associated with RAVLT learning and education was only associated with RAVLT delayed recall. ADI was not a significant predictor in any model, though the standardized coefficients were all inversely associated with memory, as expected, with absolute values comparable to demographic variables.

## 4. Discussion

Rural-dwelling older adults are underrepresented in aging research, though evidence suggests that they face greater risk for developing AD/RD. In our initial characterization of memory in the rural-dwelling cohort prospectively followed by the Nevada Exploratory Alzheimer's Disease Research Center, we found meaningful differences between urban and rural-dwelling adults on primary indices of learning, recall, and recognition for verbal list learning such that rural-dwelling older adults demonstrated a memory advantage relative to urban-dwelling adults on most measures. Given the putative risk for dementia that rural living may pose for some individuals, this was unexpected. The magnitude of advantage was small to medium in overall comparisons, but when stratified by impairment status, the magnitude of advantage was quite large for those in the cognitively normal group relative to the impaired group. In the impaired group, a small to medium rural advantage was still observed for most indices. We did not find any significant differences in learning and memory for non-verbal information, though a trend of small to medium rural advantage was again evident.

We also found that the relative magnitude of impairment was substantially larger for every measure among rural-dwellers compared to urban-dwellers. In some instances, the difference in memory between impaired and unimpaired groups in the rural cohort was double that observed in the urban cohort. As there were no differences in clinical summary measures between urban and rural cohorts, it is unlikely that the greater differences in memory are attributable to the impaired individuals in the rural cohort having more advanced disease. Given the lower levels of education in the rural impaired group, however, memory differences could reflect a lower level of cognitive reserve among some rural-dwelling older adults. Though this would not explain the memory advantage we found, it could potentially contribute to an accelerated rate of decline for older adults in a rural community once disease becomes clinically manifest and explain the greater magnitude of impairment in the rural cohort. If this is the case, this also may contribute to the higher mortality rates that have been reported (Weden et al., 2018; Rahman et al., 2021; Liu et al., 2022). Taken together, our findings suggest that late-life rural residence in this geographical region is not necessarily associated with an increased burden of memory impairment when compared cross sectionally and leaves open the possibility that late-life rural living may be protective for some individuals, especially those who intentionally choose to live in a rural community. For example, those with exceptional health and fewer cognitive symptoms may have the flexibility of choosing to live in a rural community with fewer structural and healthcare supports.

It is also possible that memory may not be the primary driver of cognitive decline in rural communities and other domains like attention, working memory, and or executive functioning may reveal more differences. Given that cardiovascular disease is more prevalent in rural communities (Aggarwal et al., 2021), a mixed dementia profile reflecting subcortical dysfunction may be evident. Not only does this warrant further investigation, this also reinforces the notion that biomarker characterization is critically important, especially in rural cohorts. Given their relatively low cost, minimal invasiveness, and ease of use, blood-based markers are well-suited for use in rural communities. In addition to studying cognitive domains beyond memory, biomarker characterization and examining the association between cardiovascular risk factors and cognition in rural older adults are key areas for further research.

Another factor to be considered is time. All individuals in the NVeADRC currently live in a rural community outside of a metropolitan area, and in the present analyses, we are only able to ascertain the effects of current rural living on memory. We are not, however, able to explore the potential influence of rural living over the life course. As recently suggested by Peterson et al. (2023), there may be differential effects of rural living for those with early- vs. late-life exposure, and for some individuals, late-life exposure may be protective. Our findings may potentially support this notion. Each individual's self-reported lifetime residential history is recorded at their initial visit for those in the NVeADRC and these data are currently being compiled, which will allow us to explore how differences in timing (e.g., early vs. middle vs. late life), dose (e.g., neighborhood disadvantage and contextual factors), and exposure (e.g., time spent in rural community) relate to cognitive outcomes in late life.

Differences in recruitment strategies in the NVeADRC and CNTN also must be considered. By actively engaging with rural-dwelling older adults directly through community outreach efforts, the cohort followed by the NVeADRC may be more representative of the broader rural Southwest communities where people live. The urban-dwelling cohort followed by the CNTN on the other hand, is more of a convenience sample followed in a clinical setting that may more closely represent individuals who have already engaged with a medical provider and are actively seeking care. In our analyses, we find that demographics and neighborhood disadvantage account for a larger proportion of variance in memory in the rural cohort compared to the urban cohort. Although in both cohorts, the overall proportion of variance explained is small, a larger portion of variance is attributed to factors beyond demographics and neighborhood disadvantage in the urban cohort, which could include clinical factors. Furthermore, more impaired rural-dwelling individuals may be less likely to participate in research, given challenges of transportation to an urban center for participation and difficulties associated with spending a day away from home. Alternatively, the lack of specialty providers in their community may encourage participation, even for those with more advanced disease. As there were no differences in overall cognitive functioning (as measured by MoCA) or functional status (as measured by CDR), and there were no differences in the prevalence of subjective memory complaints in the overall group or when stratified by impairment, differences in clinical status are an unlikely explanation. We may, however, see greater discrepancies in memory emerge if we were to follow individuals that have already established care in a rural-based clinical setting.

We also did not find any significant influence of neighborhood disadvantage on either verbal or non-verbal learning and memory. Although there is a wide range of neighborhood disadvantage in our cohorts, as measured by ADI, the relationships between neighborhood disadvantage and memory were small. Notably, the influence of neighborhood disadvantage was comparable to the influence of individual demographics on memory outcomes, reinforcing the importance of including socioeconomic context in aging research. In our present dataset, we may not have enough statistical power to evaluate the influence of neighborhood disadvantage, especially since we currently have a relatively small number of people in the most disadvantaged neighborhoods. The average level of rurality (RUCA score of 4) of the NVeADRC cohort is also a factor that may be contributing to the current results. The RUCA scale ranges from 1 to 10, with 10 being the most rural areas. Most participants live 1–2 h away from Las Vegas, and although this distance can present barriers to receiving healthcare, it may be that the disparities experienced by more rural and isolated areas are not fully represented by this cohort. While we believe that our sample is representative of older adults who currently live in rural and non-metropolitan areas in the Desert Southwest, we cannot say whether our sample is representative of the broader rural population, especially when considered from a lifetime risk/resilience perspective.

There are some notable limitations to the present study that limit generalizability. First and foremost, we do not currently have sufficient longitudinal data available that would allow us to explore the influence of rural living on aging over time. As it has been suggested that living in a rural community can increase the rate of decline (Rahman et al., 2020), this is a critical area for future study. It is also possible that the people enrolled in our cohort are able to travel independently to Las Vegas, which could bias our sample toward those with higher cognitive functioning in this cross-sectional baseline; however, over time, we expect to see different trajectories. We also have limited representation of racial and ethnic minorities in our cohort, which does not allow study of the intersection between race, ethnicity, and geography. Although rural communities in the United States are predominantly non-Hispanic white (U.S. Census Bureau, 2020), the enthoracial diversity is increasing and there are concerning trends of rising morbidity and mortality rates in rural communities (Cross and Warraich, 2021; Ho and Franco, 2022), with the greatest increases among rural Black and Hispanic communities (Cross and Warraich, 2021). We also have not yet fully characterized our cohort with AD/RD biomarkers, which is essential for identifying underlying etiology, though these data are forthcoming. We also have a limited representation of the full spectrum of AD/RD disease severity. As our cohort grows, we will be able to explore more nuanced differences between those with mild cognitive impairment and dementia. And while more rural areas (i.e., higher RUCA codes) are at greater neighborhood disadvantage, as measured by the Area Deprivation Index (ADI), disentangling differences in ADI as a function of RUCA codes is an empirical question that goes beyond the scope of the present paper, but is an important point for future inquiry.

Although rural-dwelling older adults are underrepresented in AD/RD research and critical knowledge gaps remain, the NVeADRC is working to address these gaps through prospective, longitudinal observation that includes systematic characterization of key clinical outcomes and relevant individual and contextual social determinants of health. We believe that living in a rural community presents a complex set of contextual factors that may both promote, as well as undermine, healthy aging. For many individuals, the combined influence of these exposures will increase risk for developing dementia while for others, it may promote resilience.

## Data availability statement

The raw data supporting the conclusions of this article will be made available by the authors, without undue reservation.

## Ethics statement

The studies involving humans were approved by Institutional Review Board at Cleveland Clinic. The studies were conducted in accordance with the local legislation and institutional requirements. The participants provided their written informed consent to participate in this study.

## Author contributions

JM, CW, JC, SJ, and AR contributed to the conception and design of this study. JM and JR organized the database. JM performed the statistical analysis. JM and CW wrote the first draft of the manuscript. All authors contributed to manuscript revision, read, approved the submitted version, and agree to be accountable for the content of the work.
